# Dental Education

**Published:** 1875-11

**Authors:** S. J. Cobb


### ARTICLE II.

### *Dental Education.*

BY S. J. COBB.

Read before the Tennessee Dental Association.

Having been assigned the duty of reporting upon the subject of Dental Education, I have written a few lines with a view of eliciting some thought and discussion among ourselves upon this subject, as I see that many of our best men are very much interested in the discussion of it just at this time. It seems that there is more interest manifested in the discussion of this subject at present than has been for many years; all of which I am glad to see, for I assure you, gentlemen, it is a subject that should always be uppermost in our minds.

The unusual interest felt upon the subject of dental education at present, seems to have grown out of a few editorial articles in the *Philadelphia Medical Times*—in which the editor says, "we are not entitled to the name of *Doctor*, as Dentistry in his estimation is not a specialty of medicine." Upon this subject I will say, it matters not what the *Philadelphia Times* or any other medical journal may say in regard to Dentistry as a specialty of medicine. If we continue our progression in the direction we have been going for the last quarter or half-century, we will in time satisfy the public that we are great benefactors, and in every way worthy of any name or title that we may see fit to take unto ourselves.

The fact is, we have not only proven our ability, but we are contributing largely to the alleviation of pain and suffering, as well as the restoration and improvement of man's health. If that is not doctoring, I will say it is just as good, whether it has any medicine in it or not.

In considering this part of our subject, we will ask what is medicine? Webster styles it the art of preventing, caring, or alleviating the diseases of the human body. We will now ask what is meant by the word, *Doctor?* The same author says, "It is one who has passed all the degrees of a faculty, and is empowered to practice and teach it; as a doctor in divinity, physic and law." He says a physician is a person skilled in the art of healing, one whose profession is to prescribe remedies for diseases. Taking Webster as authority upon this subject, I can hardly see how the *Times* man or any one else can refuse to give an educated dentist, one who has taken all the degrees of a faculty and is empowered to practice and teach, and in so doing, he is constantly prescribing and applying remedies for the cure and prevention of diseases, the title of doctor, or declare that he is not a specialist of medicine. If medicine is to be taken as a basis for the prevention, treatment and cure of diseases, dentistry certainly comes in for her part of the work, and in proportion to her success in preventing, treating and curing diseases, so we are entitled to the name of doctor, or specialist of medicine. I say specialist from the fact that we confine ourselves to the treatment of special organs. I am inclined to think the Editor of the *Times* bases the whole of his argument upon two or three illiberal points. The first of which is, that we have not been taught in a *medical c llege* what we know and practice. The second is, that it is hardly necessary to educate men to practice dentistry, as it can be done by ordinary mechanics. The third is, that a great many who are engaged in it, are illiterate and uneducated men. On this, the last point alone he seems to think that he has sufficient evidences to establish the truthfulness of what he has said. In a recent article he refers to Dr. James Truman, of Philadelphia, who, in discussing this subject at a meeting of the Odontological Society, he says goes as far, if not farther than he does in declaring the dental profession unworthy of being called a branch of medicine. That may all be very true, and yet fail to establish the fact that Dentistry is not a legitimate branch of medicine. The large number that Dr. Truman says are practicing without having received the degree of D. D. S., have nothing to do with the present standard of the dental profession.

It is not the masses of any profession that erects its educational standard. That is always done by the minority, and I might say a few even of that number must be held responsible for the work. So I will say if we have only a few well educated men who have gotten together and erected a standard of education that embraces the knowledge and science of treating and curing diseases of the mouth and teeth, thereby preventing other organs from becoming diseased, and in so doing assist in the preservation of the general health, we are certainly worthy of being styled a branch of medicine; that is, if medicine is to be recognized as a general basis upon which man's efforts to treat and cure disease depends. Our standard of education is as full and complete as that of the physician, as far as the treatment of such organs as we propose to treat, are concerned- and I dare say those who come up to it, are as well calcu, lated to treat successfully such organs and diseases as physicians are to treat such as they propose to treat.

The fact of our having more irregulars according to the number engaged in our business, than the physician, is no reason why we should all be wiped out.

I presume the medical profession had a larger percentage of irregulars when only forty years of age than we have at present. I insist that we shall not be wiped out on account of the large number of *irregulars* engaged in our profession. We are trying to lessen the number every year and will promise to continue our efforts in this direction until something is accomplished ; for we are sensible of the fact, that there is where the fault is. Not on our present educational standard, but in not working up to it. In regard to his second point, I will say no man or set of men can do anything well who have not been educated and properly trained to the work; especially anything of importance.

When it is understood that good sound teeth in healthy mouths add ten years to the longevity of man, to say nothing of the additional pleasure and comfort, it is not difficult to see that dentistry is a very important business, and as such necessarily requires men educated and trained to the business. No one can practice dentistry well, who has no knowledge of Anatomy, Physiology, Pathology, Therapeutics, Chemistry and Hygiene, to say nothing of the special knowledge of operative and mechanical dentistry. In addition to all these things, Materia Medica, Surgery, Microscopy, Histology and Metallurgy are necessarily taught in our dental colleges. To treat any organ or set of organs as should be done, one should know something of the whole organic structure of man, as well as the relations and functions of each organ, so as to know something of the influence of diseased organs upon healthy ones. For instance, the influence of carious teeth, and an unhealthy mouth upon the general health. With all the knowledge of the physician it remains for the dentist to explain more fully the relations of the mouth and teeth to the stomach and other organs, so as to see and understand clearly the influence of carious teeth and unhealthy mouths upon the general system. I have no doubt that there are thousands of premature deaths annually from consumption and other diseases that are traceable back to carious teeth and unhealthy mouths. The perversion of the fluid of the mouth from a healthy to a vitalized secretion is sufficient in itself to say nothing of the many aches and pains directly attributable to unsound teeth and diseased gums, to break down and destroy the equilibuum of the digestive organs, after which one is liable to almost any disease, especially where there is a predisposition.

I dare say the great number of children that die teething, would not die in the effort to get their teeth, but, from the fact that the fluid of the mouth is changed from a healthy to a vitiated secretion at that time.

These and many other things evidently show that the organs that we propose to treat are very important; as such it becomes absolutely necessary that we should be educated and properly trained for the work. A few words in regard to the first point, which is that we are not specialists of medicine, from the simple fact that we have not been taught in medical colleges how to practice dentistry. It makes no difference where a person is taught, so he is well taught to do whatever he pretends to do. We have at present nine dental colleges in the United States, besides a dental department in the Harvard University at Boston, Mass.

These colleges have been gotten up for the purpose of teaching men how to treat successfully certain organs of the system so as to promote the general health. The reason why these colleges were gotten up independent of medical colleges, is that physicians in this country thirty five or forty years ago, seemed to think themselves able to do all that was necessary for man's health. At all events, they were not disposed to divide and subdivide medicine, so we were compelled to resort to independent action, notwithstanding we proposed to treat a part of the same man the physician treats. But I am glad to say such is not the case at present; for I am satisfied that a majority of the best informed physicians in America have become convinced that the best way to advance medical science is to divide and subdivide until each department can be thoroughly mastered; an evidence of this fact was demonstrated at a meeting of the American Medical Association last year, when they opened wide the doors to specialists, by passing a resolution recognizing special practice as being legitimate.

If physicians had been as liberal forty years ago as now, or in other words if they could have seen what they now see, we would have been saved the trouble of establishing dental schools independent of medical colleges. While I am of the opinion that we have advanced more rapidly as independent specialists, I am satisfied that every department of medicine should be taught in a medical college, or rather a *medical* university. Now that we have clearly demonstrated the importance of human teeth, and the great necessity for educated scientific men to assist the people in preserving them, I anticipate no further trouble in forming a partnership with the physician for the purpose of making such arrangements as may be necessary to teach dentistry with other branches of medicine. In making such arrangements, I fear, gentlemen, we are going to have trouble among ourselves, from the fact that it may be necessary to give up the name of dental profession which I know many would not like to do, after having worked so long and hard for it. Nevertheless I believe the time is not far distant when there will be a consolidation of the two professions, which I have no doubt will, in the end, prove to be advantageous to both physician and dentist.

The fact is there never should have been any cause for an independent specialty of medicine. Everybody knows that the field of medicine is too large for any one man or set of men, as such should be divided and sub-divided until every nook and corner of the entire premises is thoroughly understood and well occupied. But in our many derisions, we must not lose sight of the importance of co-operation. All must work together if we would accomplish all that medicine is designed to do. In suggesting or advocating the idea of consolidation or merging into general medicine, I do not propose to discourage *dental college* enterprise, for I am not one of those who believe that we have more colleges at present than we need; although I must say that I think one is sufficient for any one state or city at present, and in all probability will be for the next fifty or hundred years to come. While there are two in Philadelphia, Baltimore and Boston, there are other cities in other sections of the country where dental colleges or dental departments in medical universities are needed; for instance, we have ten large states in the South, to say nothing of little Florida, where there is no college or dental department. There is again California, and the whole Pacific coast without a single dental school of any kind. A dental department in a medical university in San Francisco, California, will in a few years become an absolute necessity, and I think I can say with propriety that we need at present a dental department in the Nashville Medical University. For it seems to me that this is decidedly the best location in the south for a dental school; and now that the Nashville Medical College has consolidated with the medical department of Vanderbilt University, we hope for an endowment from that institution in case we make an effort to establish a dental department.

To show that the increased number of dental colleges in the last few years are not in the way of the older ones, and that they are adding largely to the great work that has to be done, we need only refer to such statistics as show a large increased number of students in the older colleges, and a very respectable number in the latter. The number of matriculants in the Baltimore College last session was forty-one; Pennsylvania seventy-two; Philadelphia one hundred; New York seventy; Boston twenty-four; dental department of Harvard University forty-two; Maryland, eleven ; Ohio, twenty-four; Missouri, — ; New Orleans, —

From these statistics it will be seen that the most flourishing institutions that we have, are where they are the most numerous. Competition is said to be the life of trade, and as no one doubts the truthfulness of the saying, we presume it is applicable to dental college enterprise as anything else; as such we can see the necessity for competition. While I do not advocate the necessity of establishing a college in every nook and corner in the country, I am satisfied that two or three more at present would assist very much in accomplishing the work that we propose to do. It is thought by some that we could and would be more thorough in our teaching if we had only two or three colleges, but I must say I cannot see why that should necessarily be so; for it looks to me that the two *principal points, thoroughness and numbers* would as likely be attained by competition as by endowment. But admitting the possibility of a few endowed institutions becoming more thorough in their teaching than a larger number conducted upon a self-sustaining principle, we must not ignore the fact that ten or fifteen colleges are much more likely to get at the masses who are entering our profession, than two or three endowed colleges. While thoroughness is of very great importance, it is of equal importance that we should manage, in some way so as to educate all who engage in our profession.

When we have managed to get our entire profession thoroughly interested upon this subject, we will then be better able to enforce such rules of thoroughness as we may desire.

In conclusion, I will say, that it would be well for all of our dental societies to take an active interest in this matter In this way we may after awhile get the masses of our profession pretty well interested in dental education.

---

### SELECTED ARTICLES.

